# Tolerance Assessment of *Atractylodes macrocephala* Polysaccharide in the Diet of Largemouth Bass (*Micropterus salmoides*)

**DOI:** 10.3390/antiox11081581

**Published:** 2022-08-15

**Authors:** Bo Dong, Liyun Wu, Qiaozhen Chen, Wenjie Xu, Dinggang Li, Dong Han, Xiaoming Zhu, Haokun Liu, Yunxia Yang, Shouqi Xie, Junyan Jin

**Affiliations:** 1 State Key Laboratory of Freshwater Ecology and Biotechnology, Institute of Hydrobiology, Chinese Academy of Sciences, Wuhan 430072, China; 2 University of Chinese Academy of Sciences, Beijing 100049, China; 3 Baoding Jizhong Pharmaceutical Co., Ltd., Baoding 071100, China; 4 The Innovative Academy of Seed Design, Chinese Academy of Sciences, Beijing 100101, China

**Keywords:** AMP, Nrf2/Keap1 signaling pathway, antioxidant defense, inflammation, tolerance

## Abstract

*Atractylodes macrocephala* polysaccharide (AMP) can enhance antioxidant defense and anti-inflammation, as the tolerance levels of AMP in aquaculture is important for additive utilization. However, the tolerance dose of AMP is unknown. We assess the tolerance levels of AMP in juvenile largemouth bass (3.38 ± 0.11 g) by feeding them a 0, 400, 4000, or 8000 mg/kg AMP supplemented diet for 10 weeks. The 400 mg/kg AMP dose increased growth performance. The Nrf2/Keap1 signaling pathway was activated, as indicated by Keap1 and Nrf2 protein levels in the liver. Enhanced activity of antioxidant enzymes (SOD, GPx), together with increased mRNA levels of antioxidant genes (*sod*, *gpx*) and decreased accumulation of reactive oxygen species (ROS) and MDA, was found in the liver, implying the antioxidant effect of AMP. Nutrient absorption was enhanced by AMP, as reflected by the increased length of intestinal villi and microvilli. However, 4000 and 8000 mg/kg AMP induced oxidant stress, as indicated by increased plasma ALT and AST content and decreased mRNA levels of antioxidant genes (*sod*, *gpx*) in the liver and intestinal tissues. Inflammatory reactions were also induced by high doses of AMP, as reflected by enhanced levels of pro-inflammatory cytokines (*tnfα*, *nfκb*) in the liver, intestinal, and kidney tissues and inhibited levels of anti-inflammatory cytokines (*tgfβ*, *iκb*). Histological analysis reveals inflammatory cell infiltration and tissue damage. Thus, the safe tolerance margin of AMP supplement for largemouth bass was 400–4000 mg/kg.

## 1. Introduction

Aquaculture production, worldwide, reached 114.5 million tons in 2018 [1]. However, aquaculture problems, induced by the rapid increase and high-density production of fish and other aquaculture animals, include increased disease prevalence [2]. Feed additives are added in small amounts to aquafeed for specific purposes [3]. Plant-derived feed additives, such as polysaccharides, can enhance fish growth, intestinal health, disease resistance, and stress resistance [4,5]. *Astragalus* polysaccharide supplementation enhances the immune response of *Litopenaeus vannamei* by increasing superoxide dismutase (SOD) activity and inhibiting maleic dialdehyde (MDA) content in the hemolymph [6]. HP-02 polysaccharide from honeysuckle flowers has immunomodulatory effects on on-growing common carp by reducing the expression of pro-inflammatory cytokines, such as tumor necrosis factor α (*tnfα*), and increasing the expression of anti-inflammatory cytokines, such as transforming growth factor β (*tgfβ*) [7]. *Atractylodes macrocephala* polysaccharide (AMP), the main component extracted and purified from the Chinese herbal medicine *Atractylodes macrocephala*, is studied because of its anti-tumor effect [8,9]. AMP is widely used as an additive in animal formulation for its growth-promoting effect and antioxidant effect [10]. AMP can reduce inflammatory damage and oxidative stress via the NF-κB signaling pathway [11,12]. AMP can also enhance antioxidant defense capacity by promoting the expression of glutathione peroxidase (GPx) and SOD [13,14], which are related to the Nrf2 signaling pathway [15]. However, few studies report the effects of AMP on aquatic animals.

The effects of feed additives on ingestion, growth, and the immune system are normally dose-dependent in omnivores [16,17,18,19,20]. The growth of on-growing crucian carp was increased by 40 g/kg *Enteromorpha prolifera* polysaccharide supplementation, while the opposite result was observed at a higher dose [16]. High doses of feed additives might affect intestine, liver, and kidney health. Mild damage was found in the intestinal tissues of juvenile Nile tilapia when organic trace mineral doses exceeded 50% [17]. Overdose of olaquindox, a growth-promoting feed additive, led to an increasing level of alanine aminotransferase (ALT) and aspartate aminotransferase (AST) in blood, as well as cell damage to the liver of on-growing common carp [18]. Similarly, an increase in plasma ALT and AST activities occurred when dietary *Coriolus versicolor* polysaccharides exceeded 2 g/kg in on-growing crucian carp [19]. An increase in phagocytic activity in hemocytes was only observed in *Litopenaeus vannamei* when they were fed diet supplemented with *Astragalus* polysaccharides in amounts less than 1.0 g/kg [6]. High doses of pectin and cellulose caused damage and dysfunction to the liver and intestine of juvenile yellow catfish [20]. These data show that tolerance assessment of aquatic animals to feed additives is important for the safe use of additives.

Largemouth bass (*Micropterus salmoides*) is a fast-growing aquaculture species in China, with production exceeding 0.61 million tons in 2020 [21]. However, disease is currently a major factor restricting the development of largemouth bass culture [22,23]. Functional additives are an effective way to promote growth and increase antioxidant defense in largemouth bass. Extracts of *Foeniculum vulgare* and *Artemisia annua* can enhance the specific growth rate (SGR) and the enzyme activity of SOD but decrease the MDA content [24]. Chlorogenic acid can regulate inflammatory reactions by reducing mRNA expression levels of *tnfα* [25]. Dietary sodium butyrate can improve intestinal morphology by increasing the villus width and villus height [26]. Because AMP can promote growth, antioxidant activity, and disease resistance [8,9,11,12,13,14,15], it could be used as a feed additive in largemouth bass culture. The optimal supplemental level of AMP as a feed additive for largemouth bass was determined to be 400 mg/kg without negative effects (data unpublished). However, since the maximum tolerated levels of AMP are unknown, we assessed the tolerance level of AMP supplementation in largemouth bass.

## 2. Materials and Methods

### 2.1. Experimental Diets

*Atractylodes macrocephala* polysaccharide was supplied by Baoding Jizhong Biotechnology Co., Ltd. (Hebei, China). The raw medicinal herb *Atractylodes macrocephala* was boiled three times with distilled water (1:12, *w*/*v*) for 2 h. The obtained filtrate was centrifuged. Then, the condensed filtrate was boiled with 1.0% activated carbon for 20 min, followed by standing for 12 h. The filtrate was condensed at 60 °C. After spray drying, the AMP power was obtained. The carbohydrate content of AMP was 75.0%. The content of crude protein, crude lipid, and moisture was 10.0%, 5.0%, and 5.0%, respectively. The main component of AMP was dextran. In addition, D-fructose, D-glucose, and L-arabinose were also included. Four isonitrogenous and isoenergetic diets were prepared by adding 0, 400, 4000, and 8000 mg/kg AMP to the basal diet formula of largemouth bass. The formulation and basic chemical composition are shown in Table 1. All ingredients were mixed well. After mixing with water, granular feeds with a diameter of 3 mm were prepared by a pelletizer (SLR-45, Fishery Machinery Research Institute, Chinese Academy of Fishery Sciences, Shanghai, China). The diets were dried in an oven at 70 °C for 1 h and then stored at 4 °C.

### 2.2. Feeding Trial and Sampling

The largemouth bass were obtained from Ezhou Zhenghao Fry Co., Ltd. (Ezhou, Hubei, China), and were acclimated in a recirculating aquaculture system for 2 weeks with commercial feed. Fish (3.38 ± 0.11 g) were starved for 24 h before the experiment. A total of 720 healthy fish were assigned to 24 tanks (water volume: 140 L), with six tanks for each treatment. The water temperature was kept at 25–26 °C at a pH of 7–8, dissolved oxygen > 6.8 mg/L, and NH_4_-N < 0.07 mg/L, with continuous aeration for 24 h. The fish were fed to apparent satiation twice a day (9:00 and 16:00). The experiment duration was 10 weeks.

At the end of the experiment, all fish in each tank were lightly anaesthetized with 60 mg/L MS-222 (Sigma, St. Louis, MO, USA) and weighed after starving for 12 h. Then, two fish were randomly selected from each tank for body composition analysis. Blood was taken from the caudal vein by using a syringe soaked with heparin sodium from the other two fish. The supernatant plasma was taken after a 3000× *g* centrifugation. Then, the liver, intestine, and kidney tissue samples were stored at −80 °C prior to analyses. The liver, intestine, and kidney tissues were fixed in 4% paraformaldehyde for histological analysis. The experimental protocol was approved by the ethics committee of the Institute of Hydrobiology, Chinese Academy of Sciences (approval ID: IHB20140724).

### 2.3. Chemical Composition Analysis

All chemical compositions, including moisture, crude protein, crude lipid, and ash, were analyzed according to standard methods [27]. The moisture content was determined by baking at 105 °C and then using the weight loss method for calculation. Crude protein was measured by Kjeltec Auto Analyzer 4800 (FOSS Tecator, Hoganas, Sweden). Crude lipid was determined by soxhlet extraction (Soxtec System HT Tecator, Hoganas, Sweden) with ether as a solvent. The ash content was measured after full incineration at 550 °C in a muffle furnace (Jianli Electric Furnace Factory, Hubei, China).

### 2.4. Biochemical Analysis

The biochemical analysis was conducted based on reported methods [28]. The ALT and AST in plasma and the MDA, SOD, and GPx in the liver were determined using assay kits (Nanjing Jiancheng Bioengineering Institute; Catalog: C009-2-1, C010-2-1, A003-1, A001-3, and A005-1, Jiangsu, China). The liver ROS content was also determined using an assay kit (MSKBIO Co., Ltd. Wuhan, China; Catalog: 69-86537). All the above parameters were determined according to manufacturer protocols.

### 2.5. Histological Analysis

The liver, intestine, and kidney tissues were dehydrated before paraffin embedding. They were then cut into 4-μm sections with a slicer (RM2016, Leica Instruments Co., Ltd., Shanghai, China). The sections were dyed with hematoxylin and eosin (H&E) to stain the nuclei blue and the cytoplasm red. The intestine was fixed overnight in 2.5% glutaraldehyde in 0.1 M phosphate buffer (pH = 7.4) at 4 °C and post-fixed in 1% OsO_4_ at 4 °C for 2.5 h [29]. The sections were dehydrated using graded ethanol (30, 50, 70, 90, and 100%), processed with a mixed solution of ethanol and acetone, infiltrated in a mixture of acetone and epoxy resin (1:1 for 3.5 h, and then 1:2 overnight), and finally embedded using SPI-PON 812 at 60 °C for 48 h. Ultrathin sections (74 nm) were obtained using a Leica EM UC7 ultramicrotome. The ultrathin sections were stained with 3% uranyl acetate and lead citrate and then observed and photographed with an HT7700 transmission electron microscope (Hitachi High-Tech, Tokyo, Japan). Quantitative analysis of intestinal villi was performed using Image-pro plus 6.0 software, and the microvilli were measured using Image J software.

### 2.6. RNA Extraction and Real-Time Quantitative PCR

The total RNA of the liver, intestine, and kidney tissues was extracted with TRIzol reagent (Invitrogen, Carlsbad, CA, USA). The integrity of RNA was evaluated by agarose electrophoresis. The concentration of RNA was spectrophotometrically quantified with Nanodrop 2000 (Thermo Fisher Scientific, Waltham, MA, USA). Then, the RNA was reverse transcribed into cDNA by M-MLV according to the manufacturer’s instructions. Real-time qPCR was performed on a LightCycle^®^ 480 II (Roche, Diagnostics, Basel, Switzerland) instrument with SYBR Green I Master Mix (Roche Diagnostics, Carlsbad, CA, USA). β-actin was selected as the housekeeping gene owing to its stable expression in the liver, intestine, and kidney tissues. The relative levels of target genes were calculated by the method described by Pfaffl [30]. The primers used in this experiment are listed in Table 2.

### 2.7. Western Blot

Western blot analysis of the liver tissues was performed according to the method described by Wu [31]. The primary antibodies were anti-Nrf2 (A1244, ABclonal, Wuhan, China), anti-Keap1 (A1820, ABclonal, Wuhan, China), and anti-GAPDH (ab70699, Abcam, Cambridge, UK). The bands were acquired using ImageQuant LAS 4000 mini (GE Healthcare Life Sciences, Wuxi, China) and quantified using Image J software (National Institutes of Health, Bethesda, MD, USA).

### 2.8. Statistical Analysis

All data are presented as mean ± standard error and analyzed by one-way ANOVA using SPSS Statistics 25 (International Business Machines Corp., Armonk, NY, USA). The differences were considered to be significant when *p* < 0.05.

## 3. Results

### 3.1. Growth Performance and Body Composition

The growth performance of largemouth bass is shown in Table 3. Compared with the A0 group, significantly higher FBW, WGR, SGR, and FE were found only in the A400 group. However, no significant difference was found in body composition among any of the groups.

### 3.2. Plasma Metabolites

**Figure 1:**
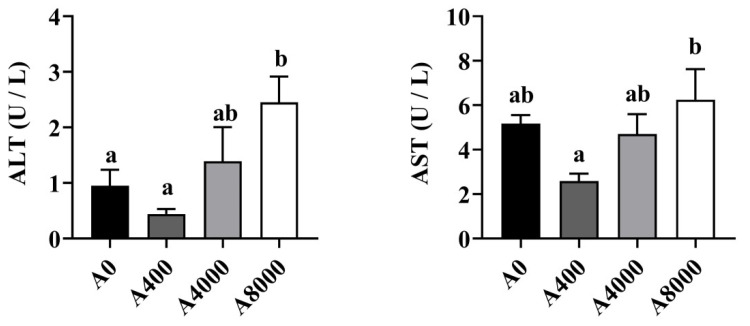
Plasma Effects of dietary *Atractylodes macrocephala* polysaccharide (AMP) on plasma ALT and AST. ALT: Alanine aminotransferase; ALT: Aspartate aminotransferase. Data are shown as mean ± SEM (*n* = 6). Different lowercase letters represent significant differences among all groups (*p* < 0.05).

The levels of ALT and AST in the plasma are shown in Figure 1. The enzyme activities of ALT and AST were significantly increased only in the A8000 group, compared to the A0 group.

### 3.3. Nrf2/Keap1 Signaling Pathway and Antioxidant-Related Genes in the Liver and Intestine

**Figure 2:**
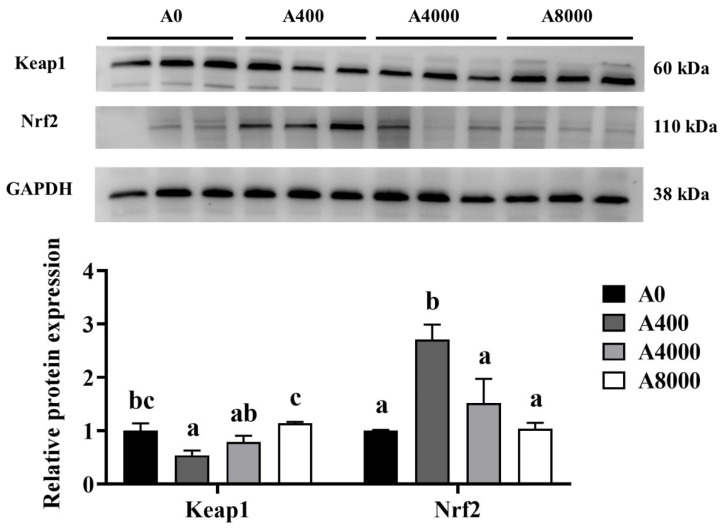
Effects of dietary AMP on protein expression and transcript levels of Keap1 and Nrf2. Keap1: Kelch-like ECH-associated protein 1; Nrf2: Nuclear factor erythroid 2-related factor 2. Data are shown as mean ± SEM (*n* = 6). Different lowercase letters represent significant differences among all groups (*p* < 0.05).

**Figure 3:**
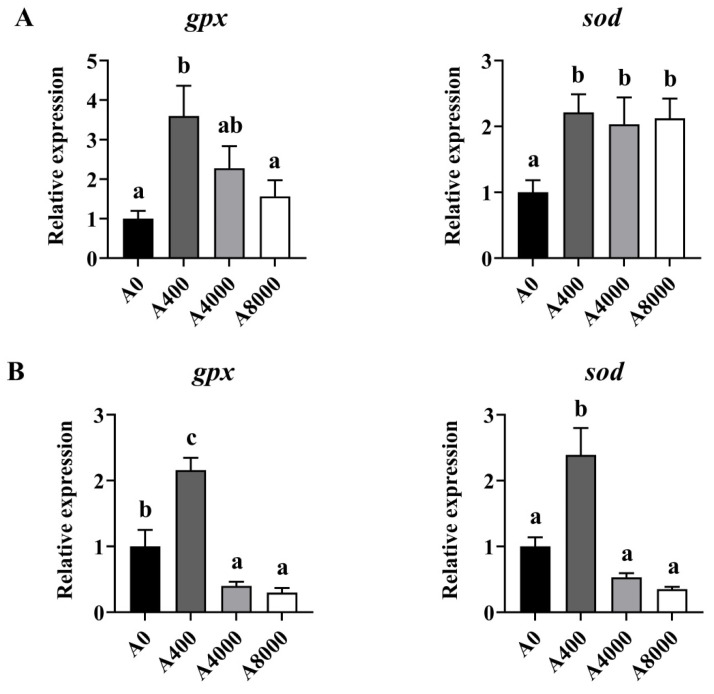
Effects of dietary AMP on expression of antioxidant-related genes in the liver (**A**) and intestine (**B**). *gpx*: glutathione peroxidase gene; *sod*: superoxide dismutase gene. Data are shown as mean ± SEM (*n* = 6). Different lowercase letters represent significant differences among all groups (*p* < 0.05).

To investigate the effect of AMP on antioxidant defense, the protein levels of Nrf2/Keap1 were determined. As shown in Figure 2, The protein levels of Keap1 in the liver were significantly inhibited by 400 mg/kg AMP supplementation; however, the opposite result was found in the 8000 mg/kg AMP supplement group. Correspondingly, Nrf2 protein expression was significantly enhanced in the A400 group compared to the A0 group. Unaltered protein levels of Nrf2 were found in both the A4000 group and the A8000 group. The expression levels of *gpx* and *sod* in both the liver and intestine were significantly induced by 400 mg/kg AMP (Figure 3A,B).

### 3.4. Activities of Antioxidant Enzymes in the Liver

**Figure 4:**
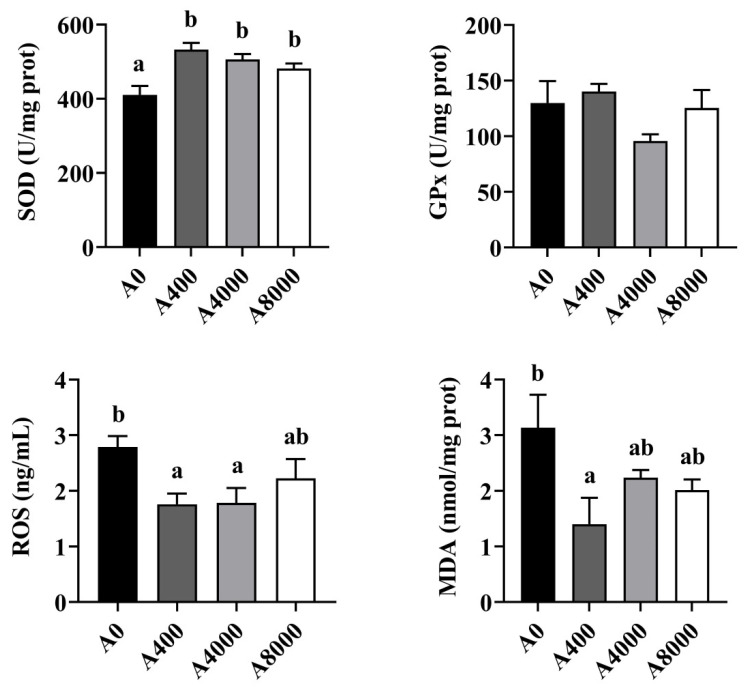
Effects of dietary AMP on activities of antioxidant enzymes in the liver (*n* = 6). SOD: superoxide dismutase; GPx: Glutathione peroxidase; ROS: Reactive oxygen species; MDA: Malondialdehyde. Data are shown as mean ± SEM (*n* = 6). Different lowercase letters represent significant differences among all groups (*p* < 0.05).

The activities of antioxidant enzymes in the liver are shown in Figure 4. The activity of SOD was increased significantly in the AMP supplemented groups, irrespective of dosage. No change of GPx was found in any group. ROS and MDA levels were significantly inhibited by 400 mg/kg AMP supplementation. However, no significant difference was found between the A0 group and the A8000 group.

### 3.5. Expression of Inflammatory-Related Genes in the Liver, Intestine, and Kidney

**Figure 5:**
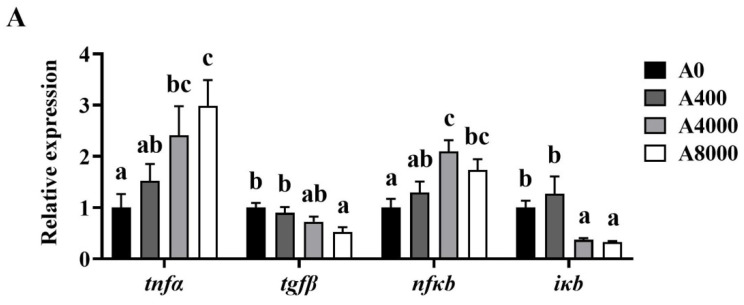

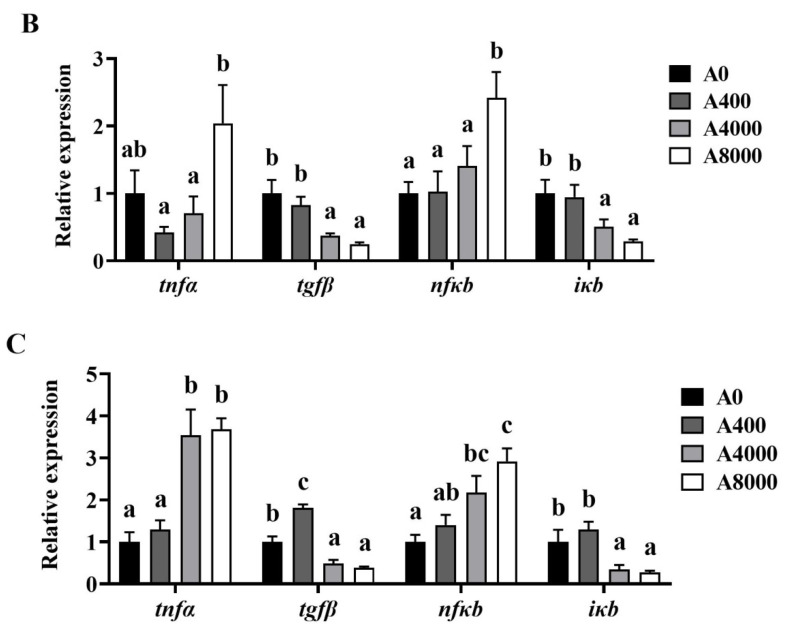
Effects of dietary AMP on expression of inflammatory-related genes in the liver (**A**), intestine (**B**), and kidney (**C**). *tnfα*: Tumor necrosis factor α; *tgfβ*: Transforming growth factor β; *nfκb*: Nuclear factor-kappa B; *iκb*: Inhibitory protein of nuclear factor-kappa B. Data are shown as mean ± SEM (*n* = 6). Different lowercase letters represent significant differences among all groups (*p* < 0.05).

Changes of the inflammation-related genes in the liver, intestine, and kidney are shown in Figure 5. The gene expression levels of *tnfα* and *nfκb* were significantly increased in the liver and kidney of the A4000 and A8000 groups, compared with the A0 group. Significantly enhanced expression of *tnfα* and *nfκb* was also found in the intestine of the A8000 group. The transcription levels of *tgfβ* and *ikb* were significantly decreased in the liver, intestine, and kidney of the A4000 and A8000 groups.

### 3.6. Histological Analysis of the Liver, Intestine, and Kidney

**Figure 6:**
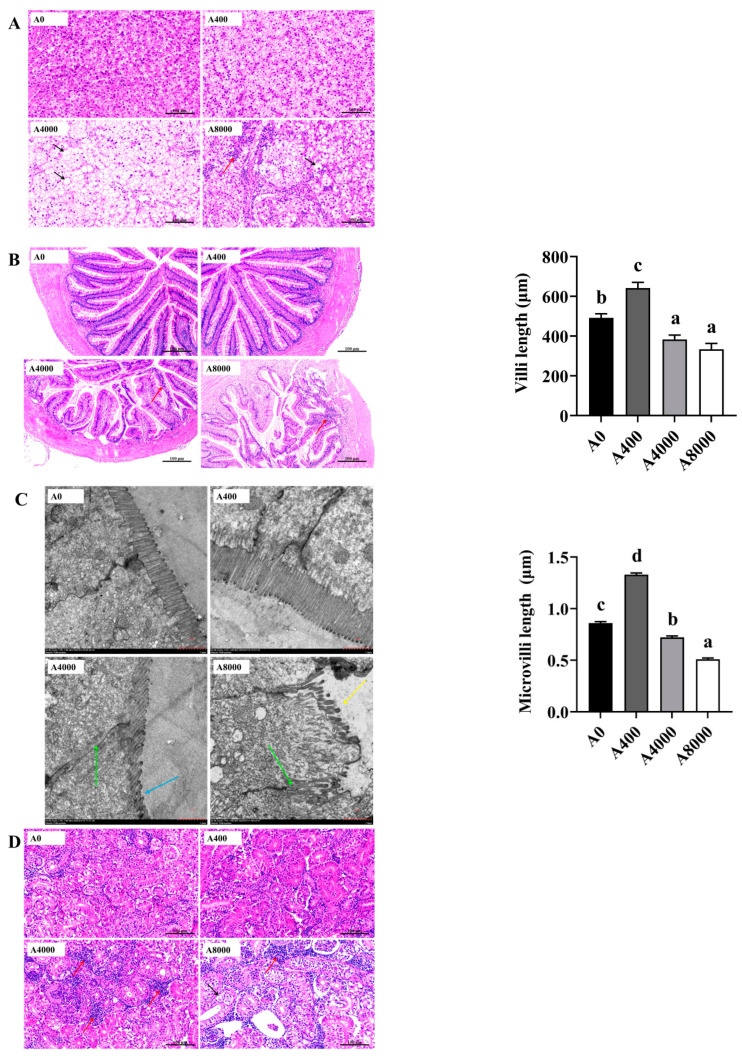
Effects of dietary AMP on histologic morphology of the liver (**A**), intestine (**B**,**C**), and kidney (**D**). Red row: inflammatory cell infiltration; black row: cell edema; green row: tight junctions; blue row: microvilli shrunk; yellow row: microvilli shed. Data are shown as mean ± SEM (*n* = 6). Different lowercase letters represent significant differences among all groups (*p* < 0.05).

The cell morphology of the liver is shown in Figure 6A. Compared with A0, no change was found in A400. However, cell edema, nucleus disappearance, and cytoplasmic vacuolation were observed in A4000. Cytoplasmic vacuolation accompanied by inflammatory cell infiltration was observed in A8000.

Figure 6B shows the intestinal morphology. A0 and A400 were relatively intact, and the goblet cells of A400 were significantly increased compared to A0. Intestinal villi in A4000 were damaged and were accompanied by inflammatory cell infiltration. Severe intestinal abnormalities and inflammatory cell infiltration were found in A8000. Compared with A0, the length of villi in A400 was significantly increased, whereas significantly decreased villi were found in A4000 and A8000. Transmission electron microscope images of villi are shown in Figure 6C. The tight junction of epithelial cells in A4000 was weakened, and the microvilli were significantly shrunken and reduced. In A8000, the intestinal wall was damaged, the tight junctions of intestinal epithelial cells were destroyed, the microvilli were wrinkled and shed, and their number was sparse. Microvillus length was significantly increased in A400 compared to A0 and was significantly decreased in A4000 compared to A0. A more severe change compared to A4000 was found in the A8000 group.

No abnormal histologic morphology was found in the kidneys of A0 and A400 (Figure 6D). A large number of inflammatory cell infiltrations were observed in the A4000 group. The renal tissue structure of A8000 was visibly abnormal, with the internal structure of glomerulus scattered and renal tubule edema accompanied by inflammatory cell infiltration.

## 4. Discussion

AMP is widely used for its antioxidant, anti-inflammatory, anti-tumor, immunopotentiator, and intestinal health maintenance benefits [8,9,11,12,13,14,15]. In the present study, 400 mg/kg AMP significantly promoted WGR, SGR, and FE. A similar growth promotion effect of polysaccharide additives was previously reported in tilapia [32], turbot [33], grey mullet [34], large yellow croaker [35], Asian seabass [36], and shrimp [37]. However, in this study, no significant difference in growth was found between fish with dietary AMP levels of 4000 mg/kg and 8000 mg/kg. These results are consistent with those on *Aloe vera* polysaccharide supplementation in African catfish [38], which might be due to higher feed additive levels affecting intestinal morphology, feed digestibility, and absorption [39,40].

ROS are active oxidants or free radicals produced by molecular oxygen gain electrons [41]. ROS can cause oxidative stress and damage to fish tissues, while antioxidant enzymes, such as SOD and GPx, can reduce ROS to protect the tissues from injury [42,43]. In the present study, 400 mg/kg dietary AMP increased the SOD activity and reduced the content of MDA and ROS in the liver. This indicates that AMP supplementation at a dosage of 400 mg/kg could increase antioxidant defense in largemouth bass. These results are consistent with those on other polysaccharide additives including *Astragalus* polysaccharide [44], *Porphyra yezoensis* polysaccharide [45], and fucoidan [46] supplementation in fish. Nrf2 can promote the expression of antioxidant-related genes after entering the nucleus, while Keap1 is a negative regulator of Nrf2 through ubiquitination and degradation and prevents Nrf2 from entering the nucleus [47]. The Nrf2 signaling pathway can regulate the expression of antioxidant-related genes and affect the antioxidant defense ability of shrimp and fish [48,49]. In this study, 400 mg/kg AMP supplementation significantly increased the expression of Nrf2 in the liver while inhibiting the expression of Keap1. Correspondingly, *sod* and *gpx* gene expression was also enhanced by 400 mg/kg AMP. Similarly, polysaccharide additives can enhance antioxidant defense in carp and tilapia by activating the Nrf2 signaling pathway [50,51]. However, the Nrf2 decreased in fish fed a diet supplemented with 4000 and 8000 mg/kg AMP, accompanied with a decrease in *sod* and *gpx* gene expression compared to 400 mg/kg AMP supplementation group. Consistent with this, the gene expression levels of nuclear factor erythroid 2-related factor 2 (*nrf2*), *sod*, and *gpx* were decreased in *Labeo rohita* fed diets supplemented with 100 mg/kg ulvan [52].

Histological analysis can reveal the functional mechanism of aquatic additives [53,54]. Here, the liver tissue was intact in the 400 mg/kg AMP supplementation group, while cell edema, nucleus disappearance, and cytoplasmic vacuolation accompanied by inflammatory cell infiltration occurred in the 4000 and 8000 mg/kg AMP supplementation groups. These results indicate that high dose (4000 and 8000 mg/kg) AMP causes severe liver injury in largemouth bass, consistent with the liver damage induced by ahigh dose of additives in common carp [18] and tilapia [55]. ALT and AST in plasma are considered as an index of liver injury in fish, which can indicate the function of the liver [56]. We found a significant increase in plasma AST and ALT in the 8000 mg/kg AMP supplementation group, which indicates that liver damage was induced by a high dose of AMP. Consistent with this, high levels of dietary histamine increased the plasma ALT and AST as well as caused hepatic injuries in yellow catfish [57]. In zebrafish (*Danio rerio*), increased villi length and surface area promote nutrient absorption [58]. Polysaccharides can also promote intestinal cell proliferation [59]. Increased length and width of intestinal microvilli promote feed digestion and absorption in Nile tilapia [60]. In the present study, the length of villi and microvilli was significantly increased in the 400 mg/kg AMP supplementation group, and this implies that nutrient absorption was increased. Supported by studies on Nile tilapia [61] and banana shrimp [62], the villus surface area and villus length and width as well as growth performance can be promoted by polysaccharide additive supplementation. However, damage to the intestinal wall and tight junctions, as well as the shrinkage and reduction in villi and microvilli, in the 4000 and 8000 mg/kg AMP supplementation groups indicates the toxic effects caused by the high concentrations of AMP on largemouth bass. Similar damaged intestinal histological morphology induced by a high dose of additives was also observed in Nile tilapia and Pacific white shrimp [17,63]. The excessive use of additives can be harmful to the kidney of fish [64,65]. Consistent with the results in the liver and intestine, tissue damage in the kidney accompanied by inflammatory cell infiltration occurred when dietary AMP levels exceeded 4000 mg/kg. These results indicate that damaged tissue integrity induced by a high dose of AMP might be the reason for reduced feed utilization and unaltered growth in these groups [66].

Inflammation occurs when innate immune cells detect infection or tissue injury [67,68]. The pro-inflammatory cytokine *tnfα* is mainly secreted by macrophages that can stimulate the secretion of interleukin-1 [69]. The anti-inflammatory cytokine *tgfβ* can inhibit the secretion of *tnfα* [70]. NF-κB (the protein translated by *nfκb* gene) activation usually induces inflammatory cytokines and adhesion molecules, which leads to the recruitment of leukocytes to inflammation sites. I-κB (the protein translated by *iκb* gene) is an inhibitor of NF-κB that promotes the degradation of NF-κB [71]. In the 400 mg/kg AMP supplementation group, the anti-inflammatory cytokine *tgfβ* was significantly increased, suggesting that AMP could enhance an anti-inflammatory response in largemouth bass. Other polysaccharides, such as *Astragalus* polysaccharide [72], *Rehmannia glutinosa* polysaccharide [73], and sulphated polysaccharide [74], also exert anti-inflammatory effects on fish. However, the expression levels of *tnfα* and *nfκb* in the liver were increased by 4000 and 8000 mg/kg AMP supplementation, while the *iκb* and *tgfβ* mRNA levels were decreased in the present study. This indicates that an inflammatory response was induced by a high dose of AMP. A similar result was found in carp [7]. Expression of pro-inflammatory cytokines, such as *tnfα*, can be promoted by high levels of HP-02 polysaccharide and induce an inflammatory response. The expression of pro-inflammatory cytokines (*tnfα*, *nfκb*) was increased and that of anti-inflammatory cytokines (*tgfβ*, *iκb*) was decreased in the intestine and kidney of fish fed with diet supplemented with 4000 or 8000 mg/kg AMP. The inflammatory cell infiltrations found in the liver, intestine, and kidney of fish fed with diet supplemented with 4000 and 8000 mg/kg AMP imply that inflammation was induced by a high dose of AMP in largemouth bass.

## 5. Conclusions

Dietary supplementation with 400 mg/kg AMP could promote the growth performance, antioxidant capacity, and intestinal health of largemouth bass. However, high doses (4000 mg/kg and 8000 mg/kg) of AMP led to decreases in antioxidant-related gene (*sod*, *gpx*) expression, accumulation of oxidative stress metabolites (ROS, MDA), inflammation inducement (upregulated pro-inflammatory cytokines *tnfα* and *nfκb*; downregulated anti-inflammatory cytokines *tgfβ* and *iκb*), and tissue damages. Villi and microvilli shrank in the intestine and cell edema occurred in the liver and kidney. Thus, the safe tolerance margin of AMP supplement for largemouth bass was 400–4000 mg/kg. This study provides valuable information for possible AMP use in aquaculture.

## Figures and Tables

**Table 1:** Formulation and composition of experimental diets (dry matter, %).

Ingredients	A0	A400	A4000	A8000
Fish meal ^a^	40	40	40	40
Wheat gluten	6.5	6.5	6.5	6.5
Casein	18.5	18.5	18.5	18.5
Flour	5	5	5	5
Cassava starch	10	10	10	10
Fish oil	6	6	6	6
Vitamin & mineral premix ^b^	1	1	1	1
Monocalcium phosphate	1.50	1.50	1.50	1.50
Choline chloride	0.10	0.10	0.10	0.10
Bentonite	11.40	11.36	11.00	10.60
AMP (mg/kg) ^c^	0	400	4000	8000
Chemical composition				
Moisture	7.91	7.97	7.16	7.28
Crude protein	52.07	52.14	51.29	51.70
Crude lipid	9.50	9.06	9.04	9.35

^a^ Fish meal: From Superprime, TASA Fish Product Co., Ltd., Lima, Peru. ^b^ Vitamin & mineral premix: P301 1% perch compound premixed feed, Yinghuier Biotechnology Co., Ltd., Beijing, China. ^c^ AMP: From Baoding Jizhong Biotechnology Co., Ltd., Hebei, China.

**Table 2:** Primers used in this experiment.

Gene Name	Sense and Antisense Primer (5′-3′)	Accession No.	Product Length (bp)
Transforming growth factor β (*tgfβ*)	ACAGTGGGCAATGTAAGCGGTA	XM_038693206.1	232
	TGTCTGGTGGGCTCTCGGTCTG		
Tumor necrosis factor α (*tnfα*)	CAAGTGTCAAACCCAGTTCCAA	XM_038723994.1	154
	ATTTGCCTCAATGTGTGACGAT		
Superoxide dismutase (*sod*)	CAGTTACCAGTGTGTCGGCTCT	XM_038727054.1	180
	CTCCAGGGCACCATAGTCGTAG		
Glutathione peroxidase (*gpx*)	CAGCAGACATTTCCTCACCATT	XM_038697220.1	250
	CAGTGGCAGAGTCAGCCTTTTA		
Inhibitory protein of nuclear factor-kappa B (*nfκb*)	GCCAGAAGACAACCATACGCAT	XM_038729519.1	164
	GGACACCAGGAGACGCTCACAC		
Nuclear factor-kappa B (*iκb*)	CACACTCGGTGATGATAACTGG	XM_038699792.1	182
	CTCCAGTAACGAGTAGTATGTA		
*β-actin*	CTTTCCTCGGTATGGAGTCTTG	MH018565.1	386
	CAGTCGTTTGGGTTTGTAGCAG		

**Table 3:** Effects of dietary AMP on growth performance.

	A0	A400	A4000	A8000
Growth performance				
IBW (%)	3.33 ± 0.06	3.40 ± 0.05	3.43 ± 0.06	3.37 ± 0.07
FBW (g)	38.47 ± 0.54 ^a^	48.62 ± 1.51 ^b^	36.76 ± 0.72 ^a^	37.64 ± 0.77 ^a^
WGR (%) ^a^	946.87 ± 60.51 ^a^	1287.39 ± 36.07 ^b^	903.13 ± 49.14 ^a^	895.64 ± 51.55 ^a^
SGR (%/d) ^b^	3.53 ± 0.02 ^a^	3.85 ± 0.05 ^b^	3.43 ± 0.03 ^a^	3.49 ± 0.03 ^a^
FE (%)^c^	119.35 ± 7.5 ^a^	146.89 ± 3.16 ^b^	114.20 ± 5.45 ^a^	112.17 ± 4.57 ^a^
Composition of whole fish				
Moisture	72.16 ± 0.17	72.09 ± 0.26	71.36 ± 1.23	72.42 ± 0.23
Ash	3.54 ± 0.05	3.60 ± 0.02	3.70 ± 0.15	3.64 ± 0.04
Crude lipid	6.87 ± 0.29	6.97 ± 0.30	7.14 ± 0.30	6.54 ± 0.17
Crude protein	16.45 ± 0.08	16.38 ± 0.08	16.89 ± 0.78	16.17 ± 0.11

The data are expressed as mean ± SEM, and the superscripts (a or b) of different letters in the same row indicate significant differences (*p* < 0.05). ^a^ WGR (weight gain rate, %) = 100 × (final total weight−initial total weight)/initial total weight. ^b^ SGR (specific growth rate, %/d) = 100 × [Ln (final body weight) − Ln (initial body weight)]/days. ^c^ FE (feed efficiency, %) = 100 × (final total weight−initial total weight)/feed intake in dry matter.

## Data Availability

Data are contained within the article.
